# Nucleos(t)ide Analog–Therapeutic Vaccination Switch: A Novel Approach for Safe and Effective Treatment Discontinuation in HBeAg (-) Chronic Hepatitis B Patients

**DOI:** 10.3390/vaccines14090821

**Published:** 2026-09-18

**Authors:** Sheikh Mohammad Fazle Akbar, Mamun Al Mahtab, Mohammad Abdur Rahim, Sheikh Mohammad Noor-E-Alam, Musarrat Mahtab, Ahmed Lutful Moben, Rokshana Begum, Dulal Chandra Das, Gerardo Guillen, Osamu Yoshida, Ivan Santos Martinez, Yoichi Hiasa, Sakirul Khan, Julio Cesar Aguilar

**Affiliations:** 1 Department of Gastroenterology and Metabology, Ehime University Graduate School of Medicine, Toon City 791-0295, Japan; yoshidao.m.ehime@gmail.com (O.Y.); hiasa@m.ehime-u.ac.jp (Y.H.); 2 Miyakawa Memorial Research Foundation, Tokyo 107-0062, Japan; 3 Graduate School of Medicine, Oita University, Oita 879-5593, Japan; sakirul.khan@gmail.com; 4 Department of Hepatology, Bangladesh Medical University, Dhaka 1000, Bangladesh; shwapnil@agni.com (M.A.M.); drnoorealam@gmail.com (S.M.N.-E.-A.); dulaldas36@gmail.com (D.C.D.); 5 Department of Hepatology, International Medical College, Gazipur 1711, Bangladesh; drrahim62@gmail.co; 6 Department of Biochemistry, Lab Science Diagnostic, Dhaka 1205, Bangladesh; musarratmahtab1999@gmail.com; 7 Department of Hepatology, Kurmitola General Hospital, Dhaka 1206, Bangladesh; mubin_dr99@yahoo.com; 8 Department of Hepatology, Shaheed Suhrawardy Medical College, Dhaka 1207, Bangladesh; rokshana.begum@gmail.com; 9 Department of Vaccines, Center for Genetic Engineering and Biotechnology, Havana 11600, Cuba; gerardo.guillen@cigb.edu.cu (G.G.); santosmartinezivanluis@gmail.com (I.S.M.); 10 Department of Pharmacy, Pharmaceutical Technology, and Physical Chemistry, University of Barcelona, 08-007 Barcelona, Spain

**Keywords:** chronic hepatitis B, NASVAC, therapeutic vaccine, NUCs, treatment discontinuation

## Abstract

Background/Objective: Long-term nucleos(t)ide analog (NUC) therapy effectively suppresses hepatitis B virus (HBV) replication, but it rarely achieves a functional cure and typically requires lifelong administration. A major unmet need in chronic hepatitis B (CHB) is the development of safe and effective strategies to discontinue NUCs without compromising viral control or hepatic safety. This exploratory, randomized pilot study evaluated therapeutic immunization in the context of discontinuation of antiviral therapy in HBeAg-negative CHB. Methods: Twenty-six HBeAg-negative CHB patients receiving long-term therapy with NUCs were randomized to either discontinue NUCs and switch to HeberNasvac (NASVAC), a therapeutic vaccine (n = 13), or receive NASVAC in addition to NUCs (n = 13). Virological, serological, biochemical, and safety parameters were assessed during a 52-week primary follow-up. In particular, ALT and AST were evaluated every 2 weeks during the first 6 months after NUC discontinuation. An extended monitoring period from weeks 96 to 192 was designed to assess virological and biochemical variables. Results: At week 48, HBV DNA remained below 2000 IU/mL in 92.3% of patients who discontinued NUCs and received NASVAC and in 100% of those who received both NUCs and NASVAC. During extended follow-up, HBV DNA < 2000 IU/mL persisted in 84.6% and 76.9% of patients in the NUC discontinuation group at weeks 96 and 192, respectively, and in 100% of patients in the continuation group (NASVAC added to NUCs) at both time points. In the first 48 weeks after NUC discontinuation, NASVAC induced a limited but significant decline in serum quantitative HBsAg. Liver function, renal, and hematological parameters remained stable, without significant ALT abnormalities, biochemical exacerbations, fibrosis progression, or severe adverse events during primary or extended follow-up. None of the patients required restarting treatment. Conclusions: NASVAC-based immunotherapy demonstrated durable virological control, a limited but progressive reduction in qHBsAg, and a strong long-term safety profile following the complete withdrawal of antiviral therapy. This exploratory study suggests that NASVAC can be administered in the context of NUC discontinuation with an acceptable short- and long-term safety profile, but controlled trials are required to determine whether therapeutic vaccination contributes independently to sustained off-treatment viral control.

## 1. Introduction

Chronic hepatitis B results in major long-term complications, particularly liver cirrhosis (LC) and hepatocellular carcinoma (HCC) [1,2,3]. Strategies that effectively prevent or slow progression to these outcomes remain central to reducing HBV-related morbidity and mortality [4,5,6].

Despite the broad implementation of preventive measures and high global vaccination coverage through the Expanded Program on Immunization (EPI), HBV-related mortality has continued to increase, now reaching 1.1 million deaths per year. The 17% rise reported in the WHO Global Hepatitis Report 2026 underscores a widening gap between current epidemiological trends and the WHO goal of achieving a 65% reduction in HBV mortality by 2030 [1].

Among the available therapeutic options, NUCs [7,8,9] have become the standard of care because of their potent ability to suppress HBV replication and reduce the risk of progression to cirrhosis and hepatocellular carcinoma. However, residual risk persists [10,11]. Treated patients rarely achieve a functional cure and fail to eliminate intrahepatic covalently closed circular DNA (cccDNA). Moreover, treatment discontinuation is frequently followed by virological relapse, hepatic flares, and a high likelihood of off-treatment recurrence [12,13]. As a result, many patients remain dependent on continuous antiviral therapy for years or even throughout life.

Experimental studies have shown that entecavir can be incorporated into mammalian DNA and induce DNA damage and chromosomal abnormalities [14,15], while long-term animal studies have reported carcinogenic effects for both entecavir and tenofovir disoproxil fumarate NUCs [16,17]. Furthermore, prolonged antiviral pressure has been associated with the emergence of mutant HBV variants with increased oncogenic potential [18]. These findings, together with the renal and bone adverse events associated with the long-term use of NUCs, highlight the need for therapeutic algorithms that allow safe treatment discontinuation and for well-defined criteria to guide retreatment in cases of virological rebound or biochemical abnormalities after cessation of NUCs.

In contrast to NUCs, which directly target viral replication, immunomodulatory treatments—and particularly therapeutic immunization—aim to restore HBV-specific immune responses and promote sustained viral control [7,8]. NASVAC is a therapeutic vaccine for nasal (IN) and subcutaneous (SC) administration comprising the hepatitis B surface and core antigens (HBsAg and HBcAg), respectively [19]. Its safety, immunogenicity, and efficacy have been demonstrated in phase I–IV clinical trials conducted in healthy volunteers and patients with chronic hepatitis B, as well as in multiple preclinical studies. Importantly, antiviral and immunomodulatory effects [20] have been shown to persist for up to five years of treatment-free follow-up [21,22].

This study assesses whether the antiviral and immunomodulatory activities of NASVAC enable a safe discontinuation of NUCs while preserving long-term virological and biochemical stability in patients with CHB. To this end, we examined the safety and preliminary efficacy of two NASVAC administration strategies: (a) NASVAC vaccination immediately following cessation of long-term therapy with NUCs; (b) NASVAC vaccination in patients on ongoing antiviral treatment.

## 2. Materials and Methods

### 2.1. Nature of the Study and Required Permission

This exploratory, unregistered randomized pilot study was conducted at Bangladesh Medical University, Dhaka, Bangladesh, in compliance with the Declaration of Helsinki and the principles of Good Clinical Practice. This trial was not prospectively registered because public disclosure of the protocol would have compromised proprietary information related to the use of a therapeutic vaccine in the context of NUC withdrawal. The study was fully approved by the Institutional Review Board (IRB No. BSMMU/2020/4329) dated 11 March 2020. All participants provided written informed consent after receiving a comprehensive explanation of the study procedures, objectives, potential risks, and their rights, in their native language.

### 2.2. Study Population

The study included CHB patients of both genders aged 18 to 65 years. All participants were HBsAg-positive and HBeAg-negative for two years, with stable treatment with NUCs for a minimum of two years before vaccination. Patients had been receiving standard-dose entecavir (0.5 or 1.0 mg/day) or tenofovir disoproxil fumarate (300 mg/day) for at least two years prior to enrolment, according to national treatment guidelines. Patients were selected with low initial viral load (≤2000 IU/mL) and stable liver function, with transaminases below 2× the upper limit of normal (ULN) during the pre-immunization assessment. The upper level of normality (ULN) for alanine aminotransferase (ALT) was defined as 35 U/L for males and 25 U/L for females, following current AASLD recommendations [23].

Patients were excluded if they had advanced liver disease with cirrhosis and/or HCC; a history of hepatitis C, hepatitis delta, or HIV infection; critical illness; uncontrolled hypertension; hyperthyroidism; epilepsy; malignancies or any uncontrolled systemic disease; pregnancy or breastfeeding; were women of childbearing potential without contraception; had known severe allergic conditions or hypersensitivity; severe psychiatric dysfunction or any limitation preventing informed consent; autoimmune diseases; or had received immunosuppressive or immunomodulatory drugs during the study or within the preceding six months. Additional exclusion criteria included a history of alcohol or drug abuse within one year prior to enrollment, other liver diseases of different etiology, and markedly elevated ALT levels at baseline (>500 U/L), suggestive of unstable disease or acute flares exceeding 10× ULN.

**Figure 1:**
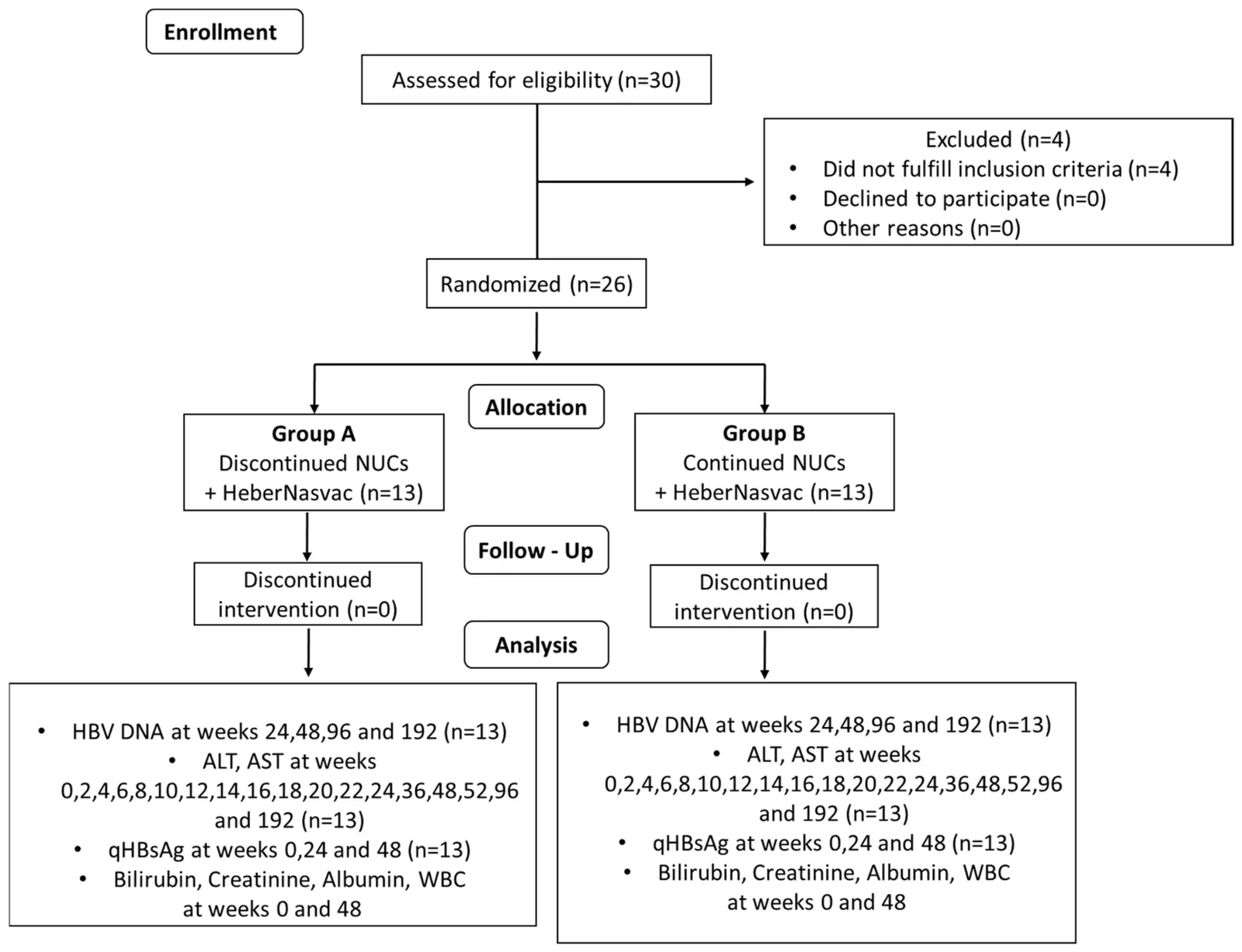
CONSORT flow diagram of patient enrollment, randomization, allocation, follow-up, and analysis. Of the 30 patients with HBeAg-negative chronic hepatitis B (CHB) assessed for eligibility, 26 met the predefined inclusion criteria and were randomly assigned in a 1:1 ratio to either discontinuation of long-term therapy with NUCs followed by NASVAC administration (n = 13) or NASVAC added to NUCs (n = 13). No patients discontinued the assigned intervention or were lost to follow-up, and all randomized participants completed the study protocol and were included in the final efficacy and safety analyses.

A total of 26 out of 30 screened CHB patients met all inclusion and exclusion criteria and were ultimately enrolled in the study. The study design is shown in Figure 1, a CONSORT flow diagram of patient enrollment, randomization, allocation, follow-up, and analysis. The trial was conducted and reported in accordance with the CONSORT 2010 guidelines. A completed CONSORT checklist is provided in Figure 1.

### 2.3. Protocol of Administration of NASVAC

**Figure 2:**
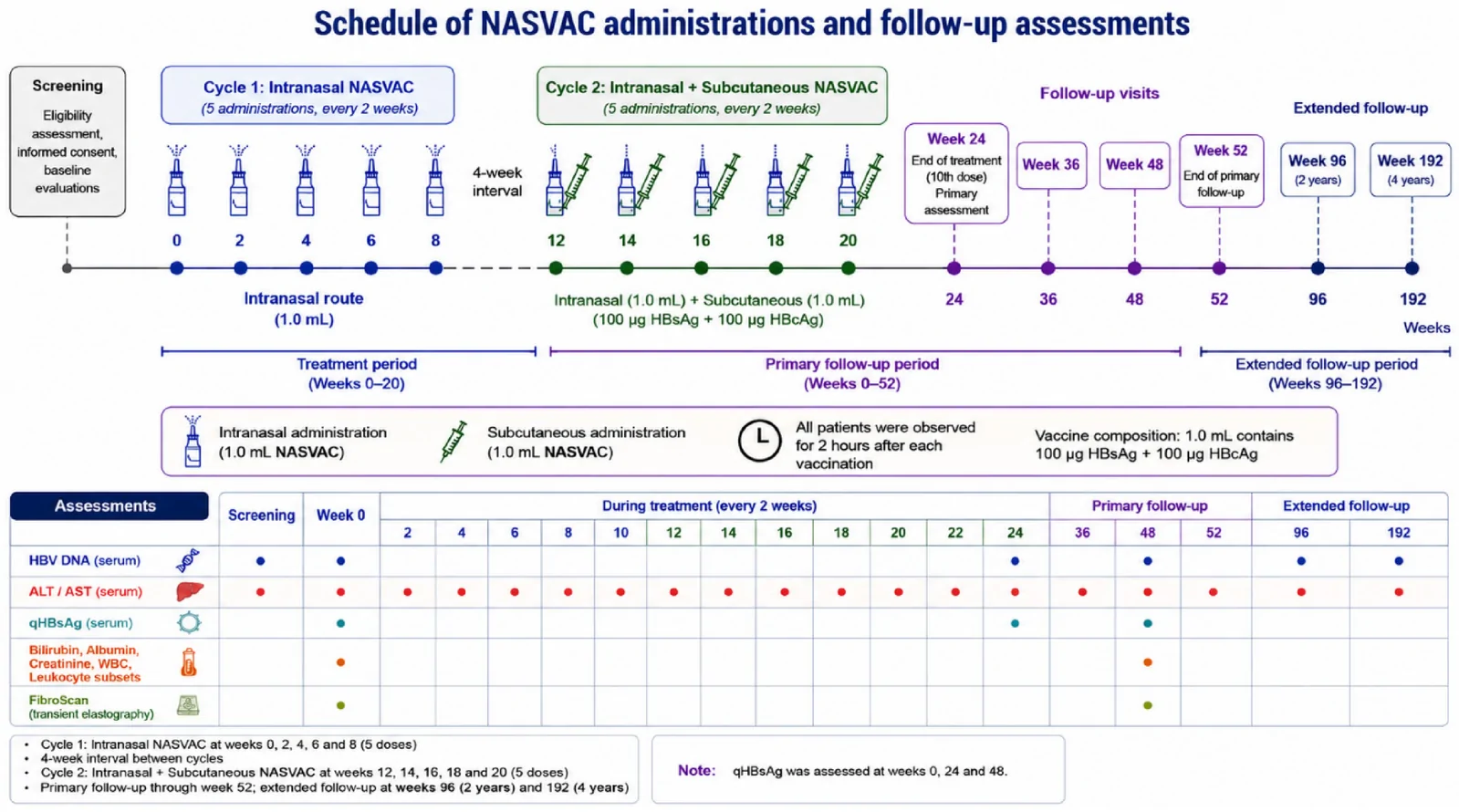
Schedule of NASVAC administration and follow-up assessments. Patients received five intranasal administrations of NASVAC at 2-week intervals during Cycle 1 (weeks 0–8), followed by a 4-week treatment-free interval and five combined intranasal plus subcutaneous administrations during Cycle 2 (weeks 12–20). Blood samples for virological, biochemical, and safety assessments were collected according to the indicated schedule, with the frequency and timing of blood extractions shown in the figure. Primary follow-up was completed through week 52, and extended follow-up was conducted at weeks 96 and 192. Both treatment groups followed the same vaccination and assessment schedule.

The patients were randomly allocated in a 1:1 ratio into two treatment groups. In Group A, long-term therapy with NUCs was discontinued, and patients were switched to therapeutic immunization with NASVAC. In Group B, patients continued their ongoing therapy with the addition of NASVAC (Figure 2).

Therapeutic immunization consisted of two treatment cycles. During the first cycle, NASVAC was administered intranasally (1.0 mL) on five occasions at 2-week intervals. The second cycle began at week 12, during which the same vaccine formulation (1.0 mL containing 100 μg HBsAg and 100 μg HBcAg) was administered intranasally and subcutaneously on five occasions at 2-week intervals. All patients were observed for 2 h after each vaccine administration.

Serum samples were collected at screening and immediately before the first NASVAC administration (week 0). During treatment, samples were obtained before each vaccine dose (every 2 weeks through week 24). Thereafter, patients underwent protocol-defined follow-up evaluations at weeks 36, 48, and 52, according to the parameter being analyzed. Additional extended follow-up assessments were performed at weeks 96 and 192 to evaluate the long-term durability of virological control and the sustained safety profile of NASVAC-based therapy.

### 2.4. Randomization

The randomization sequence was generated independently using 2N software (University of Arkansas, 2017), and allocation concealment was ensured through sequentially numbered assignments. A total of 26 HBeAg-negative CHB patients were randomly assigned to Group A or B. Patient assignment followed the order of arrival.

### 2.5. Criteria of Success

HBV DNA levels and ALT values were used as the primary outcome measures. The success criterion was defined as the absence of retreatment in any participant across the different follow-up periods, according to predefined rules for restarting NUCs.

Specifically, retreatment was indicated under any of the following conditions per EASL rules established at the time of protocol approval (2020):

- ALT values between 2 and 5× ULN persisting for more than 84 days (12 weeks), together with HBV DNA ≥ 20,000 copies/mL (≈2000 IU/mL).
- ALT values > 5× ULN persisting for ≥28 days (4 weeks).
- A confirmed and sustained ALT increase > 10× ULN, with or without associated symptoms.
- Two consecutive measurements (4-week interval) showing an increase in direct bilirubin above ULN relative to baseline, together with ALT values above ULN in the second test.
- A sustained and confirmed prolongation of prothrombin time ≥ 2.0 s above baseline, together with ALT values above ULN.

### 2.6. Flow Chart of the Study

The main purpose of this study was to assess whether NUCs can be safely and effectively discontinued in CHB patients who had received treatment for two or more years by replacing NUCs with NASVAC, an immunomodulatory therapeutic vaccine. To address this question, we evaluated patient safety during NASVAC administration and throughout a four-year follow-up period. We also examined whether the withdrawal of NUCs was associated with viral flares or biochemical exacerbations, as previously reported in NUC-treated patients.

To scientifically evaluate these aspects, patients attended the clinical trial center according to a predefined schedule. During cycle 1, NASVAC was administered intranasally at weeks 0, 2, 4, 6, and 8 (five visits). After a 4-week treatment-free interval, patients received five additional doses of NASVAC via both the intranasal and subcutaneous routes during cycle 2 at weeks 12, 14, 16, 18, and 20 (five visits). A follow-up visit was conducted four weeks after completion of the ten-dose vaccination schedule (one visit). Subsequently, patients underwent further follow-up assessments at weeks 36, 48, 96, and 192 (four visits).

In total, each CHB patient was evaluated at least 15 times for safety and additional study parameters. All follow-up visits up to week 192 were prospectively scheduled according to protocol.

### 2.7. Virological, Biochemical, and Histological Assessment

Baseline demographic and clinical variables—including age, sex, body weight, body mass index (BMI), duration of prior therapy with NUCs, baseline disease characteristics, and relevant clinical history such as toxic habits and previous treatment responses—were recorded at study entry.

Virological response was assessed by quantification of serum HBV DNA using a validated real-time PCR assay. HBV DNA measurements were performed at screening, immediately before the first NASVAC administration (week 0), and at weeks 24, 48, 96, and 192 after treatment initiation. The lower limit of detection was 2.1 IU/mL (approximately 10 copies/mL). All analyses were conducted using validated procedures and the ABI Prism 7300 SDS Real-Time PCR System (Applied Biosystems, Foster City, CA, USA).

Biochemical liver safety was evaluated through serial measurements of ALT and aspartate aminotransferase (AST) at baseline (week 0) and at weeks 2, 4, 6, 8, 10, 12, 14, 16, 18, 20, 22, 24, 36, 48, 52, 96, and 192. Patients were clinically monitored for safety and signs of liver injury every two weeks during NASVAC administration, immediately before each scheduled dose.

Additional hepatic and systemic safety parameters—including serum bilirubin, albumin, creatinine, total white blood cell (WBC) counts, and leukocyte subsets—were assessed at baseline and week 48. Quantitative serum HBsAg levels were measured at weeks 0, 24, and 48. Liver steatosis and fibrosis were evaluated by transient elastography using FibroScan^®^ (Echosens, Ivry-sur-Seine, France) at baseline and week 48 to monitor changes in hepatic status throughout the study [24,25].

### 2.8. Quantification of Serum HBsAg Levels

Serum hepatitis B surface antigen (HBsAg) concentrations were quantified using a non-competitive enzyme-linked immunosorbent assay (ELISA, FibroScan^®^ Echosens, Ivry-sur-Seine, France) based on a direct sandwich format. Serum samples from 26 patients with CHB were collected at baseline (week 0), week 24, and week 48 following initiation of NASVAC-based treatment.

ELISA microplates were coated with the anti-HBsAg monoclonal antibodies Hep1 and Hep4, which served as capture antibodies specific for HBsAg. After washing with deionized water containing Tween-20, serum samples were added and incubated for 1 h at 50 °C to allow antigen binding. Unbound material was removed by washing, and the HRP-conjugated monoclonal antibody CB. HepB4/HRP was added as the detection antibody and incubated for 1 h at 37 °C. Following additional washing steps, the enzymatic reaction was developed using o-phenylenediamine (OPD) and hydrogen peroxide as chromogenic substrates. After 30 min of incubation at room temperature (20–25 °C), optical density (OD) values were measured at 492 nm using a microplate reader.

HBsAg concentrations were calculated from a standard calibration curve and expressed as IU/mL. The qHBsAg assay had a quantification range of 10–10^4^ IU/mL and a lower limit of detection of 10 IU/mL. For statistical analyses, HBsAg values were log10-transformed. Longitudinal changes in serum HBsAg levels were evaluated relative to baseline values, and individual responses were additionally expressed as percentages of baseline HBsAg levels (baseline = 100%) for response categorization.

### 2.9. Statistics

As an exploratory pilot study, no formal sample size calculation or power analysis was performed. Normality was assessed using the Shapiro–Wilk test. Effect sizes and confidence intervals were added where appropriate. Continuous variables were expressed as mean ± standard deviation (SD) or median (interquartile range [IQR]), as appropriate, whereas categorical variables were summarized as frequencies and percentages. Given the relatively small sample size, the choice of statistical test was determined by the distribution of each variable.

Differences between treatment groups were analyzed using the unpaired Student *t*-test or the Mann–Whitney U test, according to data distribution. Comparisons between baseline (week 0) and each follow-up time point within each treatment group were performed using the paired Student *t*-test or the Wilcoxon matched-pairs signed-rank test, as appropriate. Differences in categorical variables were assessed using Fisher’s exact test.

Longitudinal changes in serum HBsAg levels were analyzed using the Friedman test followed by Dunn’s multiple-comparisons test. HBsAg concentrations were log_10_-transformed before analysis. All statistical tests were two-sided, and *p*-values < 0.05 were considered statistically significant. Statistical analyses were performed using GraphPad Prism version 9.0 (GraphPad Software, San Diego, CA, USA).

## 3. Results

### 3.1. Study Population and Baseline Characteristics

A total of 30 patients were screened; 26 met the eligibility criteria and were enrolled. Participants were randomly allocated to either Group A: NUCs switched to NASVAC (n = 13), or Group B: NASVAC added to NUCs (n = 13). All patients had HBeAg-negative chronic hepatitis B (CHB) with clinically stable disease, as confirmed by comprehensive serological, biochemical, virological, and imaging assessments—including serum HBV DNA, ALT, and AST measurements, as well as FibroScan and ultrasonographic evaluations. Prior to enrollment, all patients had received long-term therapy with NUCs for at least two years and fulfilled all predefined eligibility criteria. Baseline demographic and clinical characteristics are presented in Table 1.

Most baseline demographic and clinical variables were comparable between groups at study entry, including age, sex distribution, body weight, serum ALT and AST levels, albumin, total bilirubin, serum creatinine, and FibroScan CAP values (all *p* > 0.05). All patients had undetectable HBV DNA at baseline, reflecting sustained virological suppression under long-term therapy with NUCs. Significant differences at baseline were observed only for height, liver stiffness, and platelet count (Table 1). Overall, the two treatment groups were well balanced with respect to baseline characteristics.

### 3.2. Virological Assessment

**Figure 3:**
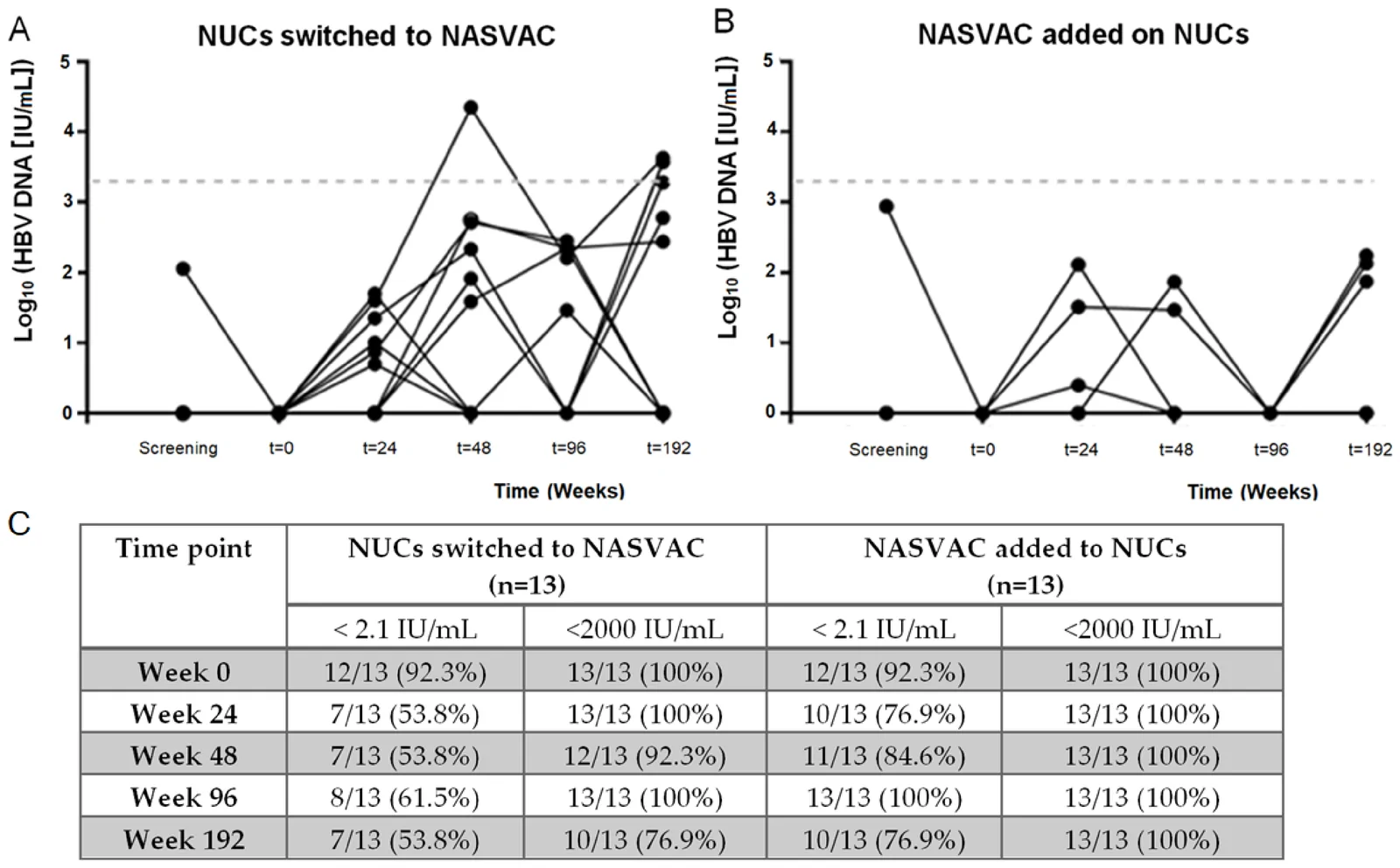
Quantitative HBV DNA values during and after NASVAC vaccination (up to week 192). (**A**) NUCs switched to the NASVAC group (n = 13). (**B**) NASVAC added to NUCs (n = 13). (**C**) Lower panel show individual serum HBV DNA levels (log_10_ IU/mL) before the first NASVAC administration (week 0) and at weeks 24, 48, 96, and 192. The dashed horizontal line represents the clinically relevant HBV DNA threshold of 2000 IU/mL (≈10^4^ copies/mL; log_10_ = 3.30).

Early virological control was evaluated by longitudinal monitoring of serum HBV DNA levels during the 48-week follow-up after NASVAC-based therapy started in both groups: Group A (NUCs switched to NASVAC treatment) and Group B (NASVAC added to NUCs) (Figure 3). At screening, HBV DNA was undetectable in 12 of 13 patients (92.3%) in each group, and repeat testing at week 0 confirmed undetectable viral loads in all participants.

At week 24, all patients remained below the clinically relevant threshold of 2000 IU/mL. Undetectable HBV DNA was observed in 7 of 13 patients (53.8%) in Group A and 10 of 13 patients (76.9%) in Group B, with no statistically significant difference between groups (*p* = 0.411). Detectable values were minimal, generally <150 IU/mL. By week 48, undetectable HBV DNA persisted in 7 of 13 patients (53.8%) in Group A and 11 of 13 patients (84.6%) in Group B (*p* = 0.202). Importantly, HBV DNA remained below 2000 IU/mL in 12 of 13 patients (92.3%) in Group A and in all patients in Group B, with only one patient in Group A exceeding this threshold during follow-up. Overall, NASVAC-based therapy maintained effective early virological control in both groups, with the vast majority of patients sustaining HBV DNA levels below 2000 IU/mL throughout the 48-week period.

Long-term virological control was evaluated at weeks 96 and 192 in both treatment groups. At week 96, undetectable HBV DNA was observed in 8 of 13 patients (61.5%) in Group A and in all 13 patients (100%) in Group B. Despite detectable HBV DNA in five patients from Group A, all remained below 2000 IU/mL. By week 192, undetectable HBV DNA persisted in 7 of 13 patients (53.8%) in Group A and in 10 of 13 patients (76.9%) in Group B. Although six patients in Group A exhibited detectable HBV DNA, half remained below 2000 IU/mL. All detectable values in Group B also remained below this threshold. Overall, HBV DNA < 2000 IU/mL was maintained in 10 of 13 patients (76.9%) in Group A and in all 13 patients (100%) in Group B at week 192. These findings indicate that NASVAC-based therapy supports durable long-term virological control, with the majority of patients in both groups maintaining HBV DNA levels below the clinically relevant threshold during extended follow-up or fluctuating near the 2000 IU/mL threshold.

### 3.3. Serological Assessment

**Figure 4:**
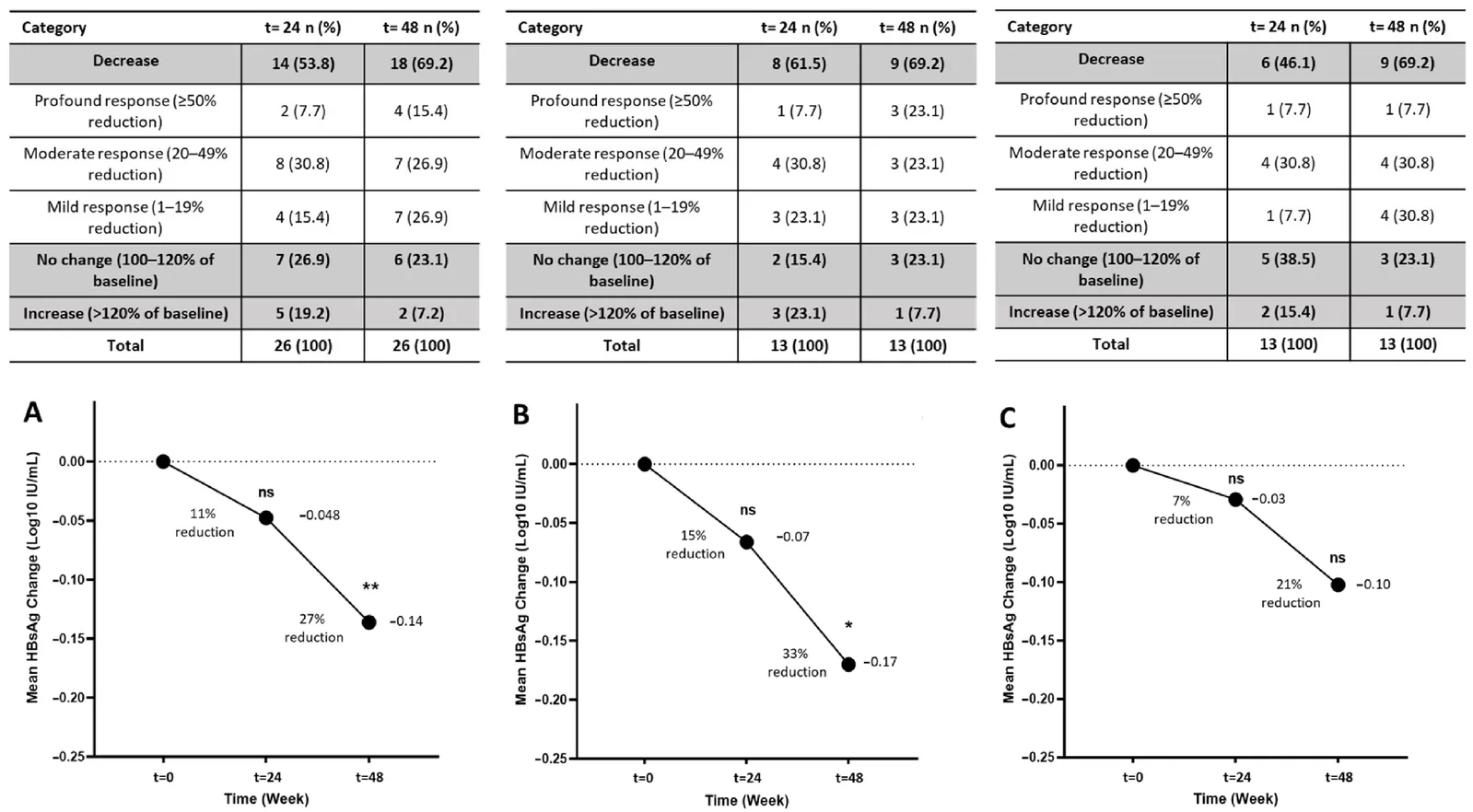
Longitudinal changes in serum HBsAg levels and distribution of response categories during the 48-week follow-up after initiation of NASVAC-based treatment. (**A**) Overall cohort (n = 26). (**B**) NUCs switched to the NASVAC group (n = 13). (**C**) Patients receiving NASVAC immunization added to NUCs (n = 13). Upper panels show mean changes in serum HBsAg levels (log_10_ IU/mL) relative to baseline at weeks 24 and 48, analyzed using the Friedman test, followed by Dunn’s multiple comparisons test. Lower panels depict the distribution of predefined HBsAg response categories at weeks 24 and 48 (baseline = 100%): profound response (≥50% reduction), moderate response (20–49% reduction), mild response (1–19% reduction), no change (100–120% of baseline), and increase (>120% of baseline). Statistical significance was defined as *p* < 0.05. ns: not significant; (*): *p* < 0.05; (**): *p* < 0.01.

Longitudinal changes in circulating HBsAg levels were evaluated over 48 weeks following NASVAC-based therapy, with values expressed as log_10_-transformed concentrations relative to baseline. In the overall cohort (n = 26), a progressive decline in serum HBsAg levels was observed (Figure 4A). Although no significant reduction was detected at week 24 (*p* = 0.8352), HBsAg levels were significantly lower by week 48 (*p* = 0.0036). A subanalysis based on baseline HBsAg levels showed that patients starting with higher antigen levels (≥500 IU/L) exhibited a markedly greater percentage reduction in qHBsAg over 48 weeks (mean 29.5%) compared with those starting <500 IU/L (mean 8%).

To characterize individual response patterns, HBsAg levels at weeks 24 and 48 were expressed as percentages of baseline. At week 24, 14 of 26 patients (53.8%) showed reductions, including two profound (≥50%), eight moderate (20–49%), and four mild (1–19%) responses. By week 48, reductions were observed in 18 of 26 patients (69.2%), with four profound, seven moderate, and seven mild responses, while only two patients (7.7%) exhibited increases. Overall, 11 of 26 patients (42.3%) achieved ≥20% reductions by week 48.

Subgroup analyses revealed distinct response dynamics (Figure 4B,C). NUCs switched to the NASVAC group showed no significant change at week 24 (*p* = 0.6949), but a significant reduction emerged by week 48 (*p* = 0.0241). The proportion of patients with reductions increased from 61.5% to 69.2%, accompanied by a rise in profound responses (7.7% to 23.1%) and a marked decline in increases (23.1% to 7.7%).

The NASVAC-plus-NUC group exhibited a gradual downward trend without reaching statistical significance at week 24 (*p* = 0.9219) or week 48 (*p* = 0.0624). Nonetheless, response distributions improved over time: reductions increased from 46.1% to 69.2%, increases declined from 15.4% to 7.7%, and mild reductions expanded from 7.7% to 30.8%.

In summary, NASVAC-based therapy induces a progressive and sustained decline in circulating HBsAg levels, with a faster decline in those discontinuing NUCs, and with both treatment strategies showing limited but favorable shifts over the 48-week period.

### 3.4. Biochemical Assessments

**Figure 5:**
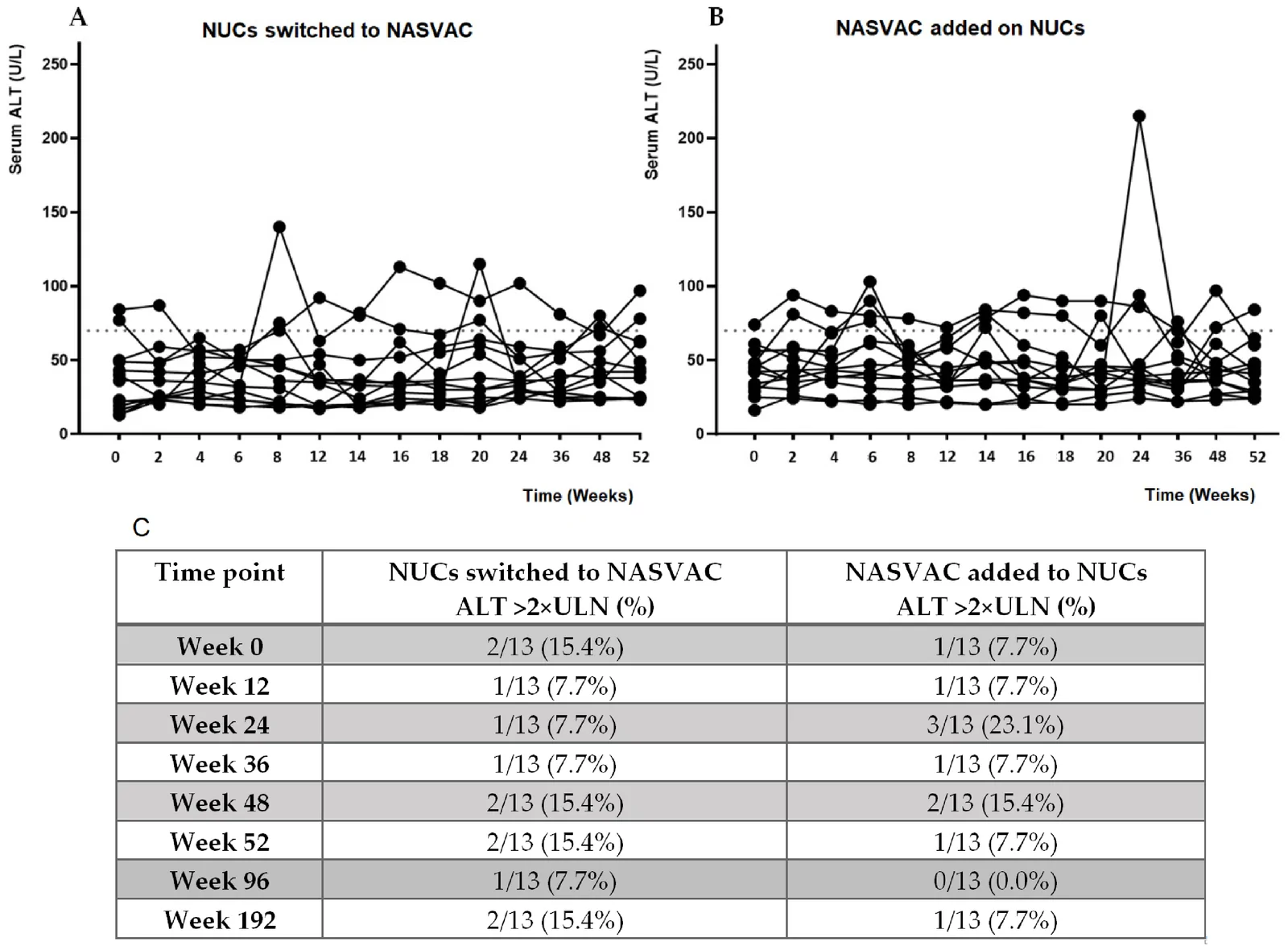
ALT levels in patients from both groups up to week 52. (**A**) Group A: NUCs switched to NASVAC. (**B**) Group B: NASVAC added to NUCs. (**C**) Proportion of ALT values above 2× ULN, including weeks 96 and 192. No significant difference was detected between groups or in the comparison of each group vs. baseline values (*p* < 0.05). The dashed horizontal line represents the clinically relevant cut off corresponding to 2 times the ULN for ALT.

Mean ALT levels remained stable over time in both treatment groups, with no significant differences. The “switch to” approach was not associated with clinically relevant biochemical abnormalities. A careful follow-up every 2 weeks during the first 24 weeks, followed by a 12-week interval in the second part of the first year, did not reveal differences between groups in either frequency or intensity (Figure 5A–C).

All follow-up visits up to week 192 were prospectively scheduled according to protocol, with minimal missing data. During extended follow-up at weeks 96 and 192, ALT levels remained stable (Figure 5C). Mean ALT concentrations were comparable between groups at both time points (*p* = 0.9680 and *p* = 0.8907), with no evidence of clinically meaningful worsening after NUC discontinuation. ALT >2× ULN remained infrequent, affecting 1 of 13 (7.7%) and 2 of 13 (15.4%) patients in the NUC discontinuation group at weeks 96 and 192, respectively, compared with 0 of 13 (0.0%) and 1 of 13 (7.7%) in Group B. No clinically relevant hepatic flares were detected during extended follow-up.

Additional hepatic function markers were evaluated by comparing serum bilirubin and albumin levels between treatment groups at baseline and week 48 (Table 2). Serum bilirubin concentrations were comparable between groups (*p* = 0.9258) and week 48 (*p* = 0.1975). Albumin levels were likewise similar between groups (baseline *p* = 0.3975; week 48, *p* = 0.5394). Albumin values remained within normal physiological ranges during the study and extended follow-up periods.

Liver steatosis and fibrosis were assessed by FibroScan at baseline and week 48 (Table 2). CAP values, reflecting hepatic steatosis, were comparable between groups at both time points (baseline *p* = 0.0592; week 48 *p* = 0.2810), with no significant within-group changes. Liver stiffness values were higher in the NASVAC-plus-NUC group at baseline and week 48, a difference already present before treatment initiation and stable throughout follow-up. Importantly, liver stiffness remained below the local cirrhosis threshold of 17.6 kPa in all patients.

Hematological and renal safety parameters—including total WBC counts, leukocyte subsets, and serum creatinine—remained stable and within normal ranges at baseline and week 48 (Table 2). No significant between-group or within-group differences were detected, and no patient exhibited clinically relevant renal dysfunction.

Overall, hepatic, renal, and hematological parameters remained stable throughout the study, supporting the safety of NASVAC-based therapeutic strategies in patients with CHB. The favorable virological and biochemical profiles observed during the primary follow-up were largely maintained during extended follow-up, with low-level HBV DNA detectability occurring in only a subset of patients after the discontinuation of NUCs and without clinically meaningful virological rebound or biochemical abnormalities.

## 4. Discussion

Long-term therapy with NUCs remains highly effective for suppressing HBV replication and preventing disease progression; however, it rarely achieves a functional cure and typically requires indefinite administration. Consequently, identifying safe and effective strategies to stop NUCs while maintaining virological control has become one of the most important unmet needs in the management of HBeAg-negative CHB [30,31,32,33].

In this study, as expected, the NASVAC-plus-NUC group showed higher rates of undetectable HBV DNA. However, the key finding is that therapeutic immunization with NASVAC enabled discontinuation of long-term NUC therapy while maintaining HBV DNA below the clinically relevant threshold of 2000 IU/mL in 92.3%, 84.6%, and 76.9% of patients at weeks 48, 96, and 192, respectively. This virological control was accompanied by stable liver enzymes with a generalized absence of clinically significant ALT abnormalities or signs of hepatic decompensation, consistent with a viral control post-cessation of NUCs.

The majority of HBV DNA values remained undetectable in both groups, with a superior but not significant proportion of patients with low-level viremia (<2000 IU/mL) in the case of patients discontinuing NUCs. Thus, Group A values are consistent with the low levels of CHB patients under effective antiviral treatment (as in Group B). Only in one case was the HBV DNA above this clinically significant cut-off (<2000 IU/mL). With this patient having normal ALT levels, retreatment was not required, overcoming the period of higher risk of viral rebound.

Previous attempts at NUC withdrawal have been limited by high relapse rates, biochemical flares, and the need for retreatment. Hadziyannis et al. reported sustained remission in 55% of HBeAg-negative patients after discontinuing adefovir [34]. A meta-analysis of 967 patients found a pooled durable response rate of 38% [35]. More recently, the large RETRACT-B study showed virological relapse in 68.9% of patients after one year and 83.4% after 48 months, with more than half requiring retreatment [36]. In comparison, most patients in the NUCs that switched to the NASVAC cohort maintained HBV DNA below treatment-relevant thresholds throughout follow-up, without requiring retreatment, suggesting the potential value of therapeutic vaccination starting at the precise timing of NUC cessation.

The absence of retreatment was used as a pragmatic safety endpoint aligned with EASL criteria but does not imply antiviral efficacy or functional cure. HBV DNA < 2000 IU/mL should be interpreted as maintenance below a clinically relevant threshold to ensure a smooth discontinuation of NUCs. However, a high proportion of patients in the NUC discontinuation group remained consistently below the lower limit of detection of the PCR assay throughout follow-up, which represents a stringent virological benchmark.

The present study provides preliminary evidence that therapeutic vaccination post-discontinuation of NUCs may be an alternative route to safely and effectively discontinue NUCs. While encouraging, these findings must be interpreted within the context of an exploratory, open-label, small-sample study that introduces a finite-treatment approach that can sustain viral control without continuous antiviral pressure and under a closely designed follow-up protocol to detect incipient signs of complications.

The favorable virological profile observed here may be related to the unique immunological properties of NASVAC. Unlike NUCs, which primarily suppress viral replication, NASVAC combines HBsAg and HBcAg to stimulate HBV-specific immune responses. Previous trials have shown that NASVAC induces antiviral and immunomodulatory effects that persist long after treatment completion [21,22,37,38]. Thus, the maintenance of viral control after discontinuation of NUCs may reflect a synergistic interaction between vaccine-induced immunity and endogenous immune activation triggered by controlled viral re-exposure; immunological studies should confirm this hypothesis in larger trials.

Another important observation was the progressive decline in quantitative HBsAg levels. Although HBsAg loss was not achieved during the primary 48-week study period, more than 40% of patients achieved ≥20% qHBsAg reductions, and distinct response patterns emerged between groups. Patients who discontinued NUC therapy before receiving NASVAC showed greater reductions in HBsAg and were the only subgroup to achieve statistically significant declines at week 48. These findings align with previous evidence suggesting that NUC discontinuation may accelerate HBsAg decline and increase the likelihood of functional cure, as shown in the FINITE and STOP-NUC trials and supported by RETRACT-B [36].

A plausible biological explanation involves the immunological consequences of antiviral withdrawal. Chronic HBV infection induces functional exhaustion of HBV-specific T cells, and although NUCs suppress viral replication, they rarely restore effective immunity [39,40]. Controlled viral re-exposure after discontinuation may provide endogenous antigenic stimulation (“auto-vaccination”), potentially reactivating HBV-specific responses [41,42,43,44]. In this context, NASVAC may amplify this effect, contributing to deeper HBsAg reductions and potentially increasing the likelihood of a functional cure.

Safety remains a critical concern in strategies for the cessation of NUCs. Severe hepatitis flares, hepatic decompensation, and even death have been reported in selected patients. In our cohort, no patient met retreatment criteria, and liver function remained stable throughout follow-up. These findings suggest that therapeutic vaccination may mitigate some of the risks traditionally associated with the withdrawal of NUCs, providing a safer framework for finite-therapy strategies.

Additional safety markers—including WBC counts, leukocyte subsets, NLR, and renal parameters—remained within normal ranges, with no evidence of systemic inflammatory deterioration. This supports the notion that viral control achieved after NUC discontinuation and NASVAC administration was not accompanied by uncontrolled or liver-damaging immune activation, an important consideration in the context of HBV-related inflammation.

This study has several limitations. The small sample size limits the ability to detect low-frequency adverse events. The open-label design introduces risks of investigator and assessment bias, although primary endpoints were objective laboratory measurements and internal comparisons represent the most relevant variables. The absence of a control group undergoing NUC discontinuation without NASVAC, as required by IRB considerations, means that differences in HBsAg kinetics between groups reflect the context of NUC discontinuation vs. continuation rather than the presence or absence of vaccination. Consequently, the independent contribution of NASVAC cannot be fully isolated. Baseline differences in liver stiffness and platelet count may influence individual trajectories and should be considered when interpreting outcomes. No immunological assays were performed, limiting the interpretations regarding immune restoration. This variable should be addressed in larger clinical trials.

Because none of our patients had qHBsAg < 50 IU/mL—the category associated with the strongest off-treatment responses in CREATE [45]—the sustained control observed in our study cannot be attributed solely to selection bias based on low qHBsAg. Although baseline antigen burden may have contributed to the overall stability observed, it does not fully explain the magnitude or consistency of the responses, and the absence of a NUC-discontinuation-only control arm remains an important limitation.

Although significant reductions in HBsAg levels were detected, clinically meaningful endpoints, such as HBsAg loss or anti-HBs seroconversion, were not observed 48 weeks after discontinuation. The reduction in qHBsAg levels will be studied in larger clinical trials across HBV genotypes, considering the low sample size and the impact of viral genotype on this variable. Efficacy results should be confirmed in a double-blind placebo-controlled phase II/III study.

## 5. Conclusions

This exploratory pilot study provides preliminary evidence supporting the feasibility of a “switch-to-vaccine” strategy as a potential approach to safely discontinuing long-term NUC therapy in patients with HBeAg-negative CHB. The sustained suppression of HBV DNA, stable ALT levels, limited but progressive decline in qHBsAg, and favorable safety profile observed after NUC withdrawal collectively support the rationale for further investigation of this strategy in larger, controlled clinical trials.

Functional cure in CHB requires durable loss of HBV DNA and HBsAg, which was not achieved during the first 48 weeks of follow-up. These findings highlight the need for extended long-term monitoring and for the evaluation of optimized vaccine formulations aimed at accelerating HBsAg decline. Future studies may explore the incorporation of potent innate immune stimulators, adjuvants, or interferon-inducing components to enhance immunological responses within this novel therapeutic scenario.

## Figures and Tables

**Table 1:** Baseline demographic and clinical characteristics of the study population.

Groups	NUCs Switched to NASVAC (n = 13)	NASVAC Added to NUCs (n = 13)	*p* Value
Number of patients	13	13	-
Age (years)	36.5 ± 9.7	37.6 ± 7.7	0.740
Sex (male/female)	10/3	12/1	0.593
Height (cm)	159.1 ± 7.2	165.6 ± 5.8	0.018 *
Weight (kg)	60 (56.5–62.5)	61 (59.5–68.5)	0.154
HBV DNA (log10 IU/mL)	Undetectable in all patients at baseline	Undetectable in all patients	-
qHBsAg (log10 IU/mL)	2.56 ± 0.69	2.46 ± 0.58	0.680
ALT (U/L)	37.3 ± 23.3	42.5 ± 15.7	0.514
AST (U/L)	30.3 ± 11.0	33.5 ± 10.0	0.441
Albumin (g/L)	47.7 ± 2.7	46.8 ± 2.8	0.397
Total bilirubin (mg/dL)	0.80 ± 0.54	0.82 ± 0.41	0.926
FibroScan CAP (dB/m)	250.5 ± 48.0	210.9 ± 53.7	0.059
Liver stiffness (kPa)	5.4 ± 1.4	9.3 ± 4.2	0.0034 **
Platelet count (× 10^9^/L)	248.9 ± 40.1	190.4 ± 63.9	0.010 *
Serum creatinine (mg/dL)	0.87 ± 0.13	0.97 ± 0.13	0.061

Note: Data are presented as mean ± standard deviation (SD), median (interquartile range [IQR]), or n (%), as appropriate. Comparisons between treatment groups were performed using the unpaired Student *t*-test, Mann–Whitney U test, or Fisher exact test, according to data distribution and variable type. All tests were two-sided, and *p* < 0.05 was considered statistically significant. (*): *p* < 0.05; (**): *p* < 0.01. Abbreviations: NASVAC, HeberNasvac; ALT, alanine aminotransferase; AST, aspartate aminotransferase; CAP, controlled attenuation parameter; HBV, hepatitis B virus; HBsAg, hepatitis B surface antigen; NUCs, nucleos(t)ide analogs.

**Table 2:** Histology, liver, and kidney function tests (bilirubin, albumin, and creatinine) and hematological evaluations during the primary 48-week period.

Parameter	Clinical Threshold (Reference)	Time Point (Weeks)	NUCs Switched to NASVAC (n = 13)	NASVAC Added to NUCs (n = 13)	Mann–Whitney/Unpaired *t* Test (*p* Value)
FibroScan (CAP)	<268 dB/m [26]	0	250.5 ± 47.9	210.9 ± 53.7	0.0592
		48	230 (198.0–291.0)	193 (190.5–262.0)	0.2810
*p* value	-	0 vs. 48	0.7364	0.5562	-
FibroScan kPa	<17.6 kPa [25]	0	5.4 ± 1.4	9.4 ± 4.2	0.0034 **
		48	5.9 ± 1.5	8.1 ± 3.3	0.0401 *
*p* value	-	0 vs. 48	0.1232	0.2896	-
Bilirubin (mg/dL)	0.2–1.2 mg/Dl [26,27]	0	0.80 ± 0.54	0.82 ± 0.41	0.9258
		48	0.60 (0.39–0.73)	0.70 (0.50–1.14)	0.1975
*p* value	-	0 vs. 48	0.2363	0.8931	-
Albumin (g/L)	35–50 g/L [26,27]	0	47.7 ± 2.7	46.8 ± 2.8	0.3975
		48	42 (39–42)	42 (40–43)	0.5394
*p* value	-	0 vs. 48	0.0002 ***	0.0001 ***	-
Creatinine (mg/dL)	0.7–1.3 mg/dL [26,27]	0	0.87 ± 0.12	0.97 ± 0.13	0.0610
		48	0.98 (0.89–1.09)	1.08 (1.03–1.11)	0.0567
*p* value	-	0 vs. 48	0.0061 **	0.0100 *	-
WBC (10^9^/L)	4.0–11.0 ×10^9^/L [26,27]	0	7.1 ± 1.7	6.2 ± 1.6	0.1776
		48	6.8 ± 1.1	6.3 ± 1.8	0.3933
*p* value	-	0 vs. 48	0.5020	0.9604	-
Neutrophils (%)	40–70% [26,27]	0	55.0 ± 6.6	63.1 ± 10.4	0.0259 *
		48	60.5 ± 9.1	68.7 ± 8.3	0.0247 *
*p* value	-	0 vs. 48	0.0133 *	0.0478 *	-
Lymphocytes (%)	20–45% [26,27]	0	36.5 ± 5.9	29.7 ± 8.7	0.0279 *
		48	27.9 ± 6.5	23.4 ± 6.9	0.1015
*p* value	-	0 vs. 48	<0.0001 ***	0.0205 *	-
NLR	0.78–3.53 [28,29]	0	1.6 ± 0.5	2.4 ± 0.9	0.0130 *
		48	2.0 (1.7–2.8)	2.9 (2.4–4.3)	0.0387 *
*p* value	-	0 vs. 48	0.0005 ***	0.0403 *	-
Monocytes (%)	2–10% [26,27]	0	4.39 ± 1.61	4.39 ± 1.56	>0.9999
		48	5.0 (4.0–6.0)	5.0 (4.0–5.5)	0.4378
*p* value	-	0 vs. 48	0.3041	0.8716	-
Eosinophils (%)	1–6% [26,27]	0	3.0 (2.0–5.0)	2.0 (1.0–4.0)	0.0952
		48	4.0 (2.5–7.5)	3.0 (2.0–5.0)	0.1371
*p* value	-	0 vs. 48	0.0859	0.0938	-

Note: Data are presented as mean ± standard deviation (SD) for normally distributed variables and as median (interquartile range, IQR) for non-normally distributed variables. Reference intervals were based on standard adult clinical laboratory ranges [25,26,27,28,29]. Abbreviations: CAP, controlled attenuation parameter; IQR, interquartile range; NLR, neutrophil-to-lymphocyte ratio; NUCs, nucleos(t)ide analogs; SD, standard deviation; WBC, white blood cells. Differences between treatment groups (Mann–Whitney U test); comparisons between baseline (week 0) and each follow-up time point (Wilcoxon matched-pairs signed-rank test/unpaired *t* test).

## Data Availability

The data and questionnaires that support the findings of this study are available from the corresponding authors upon reasonable request.

## References

[1] World Health Organization (2026). Global Hepatitis Report 2026.

[2] Seto W.K., Lo Y.R., Pawlotsky J.M., Yuen M.F. (2018). Chronic hepatitis B virus infection. Lancet.

[3] Devarbhavi H., Asrani S.K., Arab J.P., Nartey Y.A., Pose E., Kamath P.S. (2023). Global burden of liver disease: 2023 update. J. Hepatol..

[4] Cornberg M., Sandmann L., Jaroszewicz J., Kennedy P., Lampertico P., Lemoine M., Lens S., Testoni B., Wong G.L.H., Russo F.P. (2025). EASL Clinical Practice Guidelines on the management of hepatitis B virus infection. J. Hepatol..

[5] Lok A.S.F. (2024). Toward a functional cure for hepatitis B. Gut Liver.

[6] Marrapu S., Kumar R. (2024). Chronic hepatitis B: Prevent, diagnose, and treat before the point of no return. World J. Hepatol..

[7] Wong G.L.H., Gane E., Lok A.S.F. (2022). How to achieve functional cure of HBV: Stopping NUCs, adding interferon or new drug development?. J. Hepatol..

[8] Lim S.G., Baumert T.F., Boni C., Ganet E., Levrero M., Lok A.S., Maini M.K., Terrault N.A., Zoulim F. (2023). The scientific basis of combination therapy for chronic hepatitis B functional cure. Nat. Rev. Gastroenterol. Hepatol..

[9] Jeng W.J., Lok A.S. (2024). How to achieve a functional cure for chronic hepatitis B infection. Clin. Liver Dis..

[10] Moriyama M., Tateishi R., Kinoshita M.N., Fukumoto T., Yamada T., Wake T., Nakagomi R., Nakatsuka T., Minami T., Sato M. (2023). Impact of nucleos(t)ide analogues on the risk of hepatocellular carcinoma in chronic hepatitis B patients: A time-dependent Cox regression analysis. medRxiv.

[11] Wong G.L., Lampertico P. (2019). Residual risk of HCC during long-term oral nucleos(t)ide analogs (NUCs) in patients with CHB—Is one NUC better than the other?. J. Hepatol..

[12] Kuo M.T., Hu T.H., Hung C.H., Wang J.H., Lu S.N., Tsai K., Chen C.H. (2019). Hepatitis B virus relapse rates in chronic hepatitis B patients who discontinue either entecavir or tenofovir. Aliment. Pharmacol. Ther..

[13] van Bömmel F., Stein K., Heyne R., Petersen J., Buggisch P., Berg C., Zeuzem S., Stallmach A., Sprinzl M., Schott E. (2023). A multicenter randomized-controlled trial of nucleos(t)ide analogue cessation in HBeAg-negative chronic hepatitis B. J. Hepatol..

[14] Brown J.A., Pack L.R., Fowler J.D., Suo Z. (2012). Pre-steady-state kinetic investigation of the incorporation of anti-hepatitis B nucleotide analogs catalyzed by noncanonical human DNA polymerases. Chem. Res. Toxicol..

[15] Jiang L., Wu X., He F., Liu Y., Hu X., Takeda S., Qing Y. (2016). Genetic evidence for genotoxic effect of entecavir, an anti-hepatitis B virus nucleotide analog. PLoS ONE.

[16] Bristol-Myers Squibb Canada (2020). Baraclude (Entecavir) Tablets 0.5 mg, Antiviral. Product Monograph. https://www.bms.com/assets/bms/ca/documents/productmonograph/BARACLUDE_EN_PM.pdf.

[17] Gilead Sciences Inc. (2012). Viread (Tenofovir Disoproxil Fumarate) Tablets: 150, 200, 250 and 300 mg; Oral Powder: 40 mg per 1 g Oral Powder. Prescribing Information. https://www.accessdata.fda.gov/drugsatfda_docs/label/2012/022577lbl.pdf.

[18] Lin C.L., Chu Y.D., Yeh C.T. (2019). Emergence of oncogenic-enhancing hepatitis B virus X gene mutants in patients receiving suboptimal entecavir treatment. Hepatology.

[19] Aguilar J.C., Lobaina Y., Muzio V., Garcia D., Penton E., Iglesias E., Pichardo D., Urquiza D., Rodríguez D., Silva D. (2004). Development of a nasal vaccine for chronic hepatitis B infection that uses the ability of hepatitis B core antigen to stimulate a strong Th1 response against hepatitis B surface antigen. Immunol. Cell Biol..

[20] Aguilar J.C., Aguiar J., Akbar S.M.F. (2022). Action mechanisms and scientific rationale of using nasal vaccine (HeberNasvac) for the treatment of chronic hepatitis B. Vaccines.

[21] Al Mahtab M., Akbar S.M.F., Aguilar J.C., Guillen G., Penton E., Tuero A., Yoshida O., Hiasa Y., Onji M. (2018). Treatment of chronic hepatitis B naïve patients with a therapeutic vaccine containing HBs and HBc antigens (a randomized, open and treatment controlled phase III clinical trial). PLoS ONE.

[22] Al Mahtab M., Akbar S.M.F., Aguilar J.C., Yoshida O., Khan S., Getrardo G.N., Hialsa Y. (2023). Safety profile, antiviral capacity, and liver protection of a nasal therapeutic vaccine in patients with chronic hepatitis B: Five-year follow-up outcomes after the end of treatment. Front. Med..

[23] Terrault N.A., Lok A.S.F., McMahon B.J., Chang K.-M., Hwang J.P., Jonas M.M., Brown R.S., Bzowej N.H., Wong J.B. (2018). Update on prevention, diagnosis, and treatment of chronic hepatitis B: AASLD 2018 hepatitis B guidance. Hepatology.

[24] Foucher J., Chanteloup E., Vergniol J., Castéra L., Le Bail B., Adhoute X., Bertet J., Couzigou P., de Lédinghen V. (2006). Diagnosis of cirrhosis by transient elastography (FibroScan): A prospective study. Gut.

[25] Karlas T., Petroff D., Sasso M., Fan J.G., Mi Y.Q., de Léédinghen V., Kumar M., Lupsor-Platon M., Han K.H., Cardoso A.C. (2017). Individual patient data meta-analysis of controlled attenuation parameter (CAP) technology for assessing steatosis. J. Hepatol..

[26] Burtis C.A., Bruns D.E. (2014). Tietz Fundamentals of Clinical Chemistry and Molecular Diagnostics.

[27] McPherson R.A., Pincus M.R. (2021). Henry’s Clinical Diagnosis and Management by Laboratory Methods.

[28] Zahorec R. (2021). Neutrophil-to-lymphocyte ratio, past, present and future perspectives. Bratisl. Lek. Listy.

[29] Forget P., Khalifa C., Defour J.P., Latinne D., Van Pel M.C., De Kock M. (2017). What is the normal value of the neutrophil-to-lymphocyte ratio?. BMC Res. Notes.

[30] Lok A.S., McMahon B.J., Brown R.S., Wong J.B., Ahmed A.T., Farah W., Almasri J., Alahdab F., Benkhadra K., Mouchli M.A. (2016). Antiviral therapy for chronic hepatitis B viral infection in adults: A systematic review and meta-analysis. Hepatology.

[31] Wu C.-Y., Lin J.-T., Ho H.J., Su C.-W., Lee T.-Y., Wang S.-Y., Wu C., Wu J.-C. (2014). Association of nucleos(t)ide analogue therapy with reduced risk of hepatocellular carcinoma in patients with chronic hepatitis B: A nationwide cohort study. Gastroenterology.

[32] Hsu Y.C., Tseng C.H., Kao J.H. (2023). Safety considerations for withdrawal of nucleos(t)ide analogues in patients with chronic hepatitis B: First, do no harm. Clin. Mol. Hepatol..

[33] Broquetas T., Carrión J.A. (2022). Current perspectives on nucleos(t)ide analogue therapy for the long-term treatment of hepatitis B virus. Hepat. Med..

[34] Hadziyannis S.J., Sevastianos V., Rapti I.N., Vassilopoulos D., Hadziyannis E. (2006). Sustained biochemical and virological remission after discontinuation of 4 to 5 years of adefovir dipivoxil (ADV) treatment in HBeAg-negative chronic hepatitis B. Hepatology.

[35] Papatheodoridis G., Vlachogiannakos I., Cholongitas E., Wursthorn K., Thomadakis C., Touloumi G., Petersen J. (2016). Discontinuation of oral antivirals in chronic hepatitis B: A systematic review. Hepatology.

[36] Hirode G., Choi H.S., Chen C.H., Su T.H., Seto W.K., Van Hees S., Papatheodoridi M., Lens S., Wong G., Brakenhoff S.M. (2022). Off-therapy response after nucleos(t)ide analogue withdrawal in patients with chronic hepatitis B: An international, multicenter, multiethnic cohort (RETRACT-B study). Gastroenterology.

[37] Akbar S.M.F., Al Mahtab M., Aguilar J.C., Yoshida O., Khan S., Penton E., Gerardo G.N., Hiasa Y. (2021). The safety and efficacy of a therapeutic vaccine for chronic hepatitis B: A follow-up study of phase III clinical trial. Vaccines.

[38] Yoshida O., Akbar S.M.F., Imai Y., Sanada T., Tsukiyama-Kohara K., Kamishita T., Miyake T., Tokumoto Y., Hikita H., Tsuge M. (2023). Intranasal therapeutic vaccine containing HBsAg and HBcAg for patients with chronic hepatitis B: 18-month follow-up results of a phase IIa clinical study. Hepatol. Res..

[39] Boni C., Fisicaro P., Valdatta C., Amadei B., Di Vincenzo P., Giuberti T., Laccabue D., Zerbini A., Cavalli A., Missale G. (2007). Characterization of hepatitis B virus (HBV)-specific T-cell dysfunction in chronic HBV infection. J. Virol..

[40] Das A., Hoare M., Davies N., Lopes A.R., Dunn C., Kennedy P.T., Alexander G., Finney H., Lawson A., Plunkett F.J. (2008). Functional skewing of the global CD8 T-cell population in chronic hepatitis B virus infection. J. Exp. Med..

[41] Berg T., Simon K.-G., Mauss S., Schott E., Heyne R., Klass D.M., Eisenbach C., Welzel T.M., Zachoval R., Felten G. (2017). Long-term response after stopping tenofovir disoproxil fumarate in non-cirrhotic HBeAg-negative patients: FINITE study. J. Hepatol..

[42] Rinker F., Zimmer C.L., Höner Zu Siederdissen C., Manns M.P., Kraft A.R.M., Wedemeyer H., Björkström N.K., Cornberg M. (2018). Hepatitis B virus-specific T cell responses after stopping nucleos(t)ide analogue therapy in HBeAg-negative chronic hepatitis B. J. Hepatol..

[43] Höner zu Siederdissen C., Rinker F., Maasoumy B., Wiegand S.B., Filmann N., Falk C.S., Deterding K., Port K., Mix C., Manns M.P. (2016). Viral and host responses after stopping long-term nucleos(t)ide analogue therapy in HBeAg-negative chronic hepatitis B. J. Infect. Dis..

[44] van Bömmel F., Berg T. (2021). Risks and Benefits of Discontinuation of Nucleos(t)ide Analogue Treatment: A Treatment Concept for Patients With HBeAg-Negative Chronic Hepatitis, B. Hepatol. Commun..

[45] Sonneveld M.J., Park J.Y., Kaewdech A., Seto W.K., Tanaka Y., Carey I., Papatheodoridi M., van Bömmel F., Berg T., Zoulim F. (2022). Prediction of sustained response after nucleo(s)tide analogue cessation using HBsAg and HBcrAg levels: A multicenter study (CREATE). Clin. Gastroenterol. Hepatol..

